# DNA damage signalling histone H2AX is required for tumour growth

**DOI:** 10.1038/s41420-024-01869-9

**Published:** 2024-02-24

**Authors:** Lizbeth Contreras, Lorena García-Gaipo, Berta Casar, Alberto Gandarillas

**Affiliations:** 1 Cell cycle, Stem Cell Fate and Cancer Laboratory, Institute for Research Marqués de Valdecilla (IDIVAL), 39011 Santander, Spain; 2 Instituto de Biomedicina y Biotecnología de Cantabria (IBBTEC), Consejo Superior de Investigaciones Científicas (CSIC)-Universidad de Cantabria (UC), 39011 Santander, Spain; 3 Centro de Investigación Biomédica en Red de Cáncer (CIBERONC), Instituto de Salud Carlos III, 28029 Madrid, Spain; 4 Institut National de la Santé et de la Recherche Médicale, (INSERM), Délégation Occitanie, 34394 Montpellier, France

**Keywords:** Cancer, Disease model

## Abstract

Cancer most frequently develops in self-renewal tissues that are the target of genetic alterations due to mutagens or intrinsic DNA replication errors. Histone γH2AX has a critical role in the cellular DNA repair pathway cascade and contributes to genomic stability. However, the role of γH2AX in the ontology of cancer is unclear. We have investigated this issue in the epidermis, a self-renewal epithelium continuously exposed to genetic hazard and replication stress. Silencing H2AX caused cell cycle hyperactivation, impaired DNA repair and epidermal hyperplasia in the skin. However, mutagen-induced carcinogenesis was strikingly reduced in the absence of H2AX. KO tumours appeared significantly later than controls and were fewer, smaller and more benign. The stem cell marker Δp63 drastically diminished in the KO epidermis. We conclude that H2AX is required for tissue-making during both homoeostasis and tumourigenesis, possibly by contributing to the control and repair of stem cells. Therefore, although H2AX is thought to act as a tumour suppressor and our results show that it contributes to homeostasis, they also indicate that it is required for the development of cancer.

## Introduction

Both healthy tissue development and carcinogenesis need the support of cell growth. Essential barriers between one and another are the cell cycle checkpoints and the DNA repair pathways. They ensure the control of cell growth even upon mutagenic insult. This is more critical in self-renewal tissues, subjected to DNA replication stress, furthermore when they are continuously exposed to mutagenic hazard. Human mutations in the DNA repair machinery, such as xeroderma pigmentosa (XP) demonstrate how essential this process is, as patients develop skin tumours when exposed to daylight [1].

The DNA damage response pathways (DDR) rapidly activate a cascade of proteins to drive DNA repair and concomitantly trigger cell cycle checkpoints to allow cells time to repair [2]. A central signal of the DDR is γH2AX [3, 4]. Kinases ATR, ATM and DNAPK recognise alterations in the DNA chain and activate H2AX by phosphorylation (γH2AX). In turn, γH2AX recruits into the lesion a group of other proteins that control and achieve DNA repair.

γH2AX is involved in the maintenance of genomic stability due to its central role in the DDR [4, 5], critical in order to avoid transmitting cellular mutations to the progeny that might cause disease. Genomic instability is one of the hallmarks of cancer [6]. γH2AX also constitutes a cell cycle break, because it activates pathways involved in the cell cycle checkpoints that halt cell division to allow DNA repair. Therefore, H2AX is considered an anti-oncogenic barrier and has been proposed as a tumour suppressor [7, 8]. Loss of the γH2AX signal has been found in a variety of cancers [7, 9, 10]. However, attempts to identify this histone as a prognostic marker in human cancer have been inconclusive [11, 12]. In addition, tumourigenesis has not been tested in the absence of H2AX. The mere inactivation of the histone in vivo does not cause tumours unless the tumour suppressor p53 is also deleted [13, 14]. Therefore, the role of H2AX in tumour progression is still intriguing.

Stratified epithelia are continuously developing tissues greatly exposed to environmental mutagens. In stratified epithelial cells of the skin or the head and neck, unrepaired DNA damage induces terminal differentiation via mitotic checkpoints [15, 16]. This response constitutes an anti-tumour mechanism, as it blocks mitosis and maintains homoeostasis even upon cell cycle hyperactivation and replication stress [17]. We have proposed this DNA-induced differentiation response as an automatic mechanism that maintains the integrity of the tissue and at the same time, cleanses it of damaged precancerous cells. There is also evidence suggesting that the DDR pathways drive the epidermoid phenotype, even in the lung, thus controlling epithelial cell fate [16, 18].

We now have studied the consequences of silencing H2AX in skin self-renewal and carcinogenesis in vivo. To our knowledge, this is the first report studying tumour progression in the absence of H2AX. We found that epidermal progenitors more actively cycled in the absence of H2AX and had a higher capacity to amplify. Surprisingly, skin cancer is strikingly impaired. The results indicate that the lack of H2AX alleviates a cell cycle brake causing skin hyperplasia, but at the same time, triggers the squamous differentiation signals due to a higher index of unrepaired DNA damage. As a result, the growth of the tumour tissue is compromised. The findings suggest a dual role for H2AX as part of the DDR, in homeostasis and carcinogenesis. We therefore propose that caution is needed in order to consider H2AX as a tumour suppressor.

## Results

**Fig. 1:**
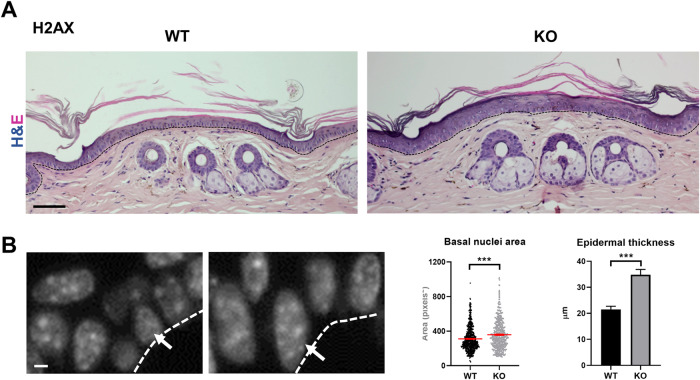
H2AX KO epidermis is hyperplastic. **A** Hematoxylin/Eosin staining of the skin of wild type (WT) or KO epidermis, as indicated. **B** Amplified basal nuclei of WT or KO epidermis stained by DAPI. Histograms: left, size of basal nuclei; right, average thickness of epidermis. Scale bar: top: 120 μm. bottom: 20 μm.

To investigate the role of H2AX in the continuous proliferation that occurs in self-renewal, we studied the epidermis of H2AX KO mice [4] as compared to control littermates. First, we confirmed the lack of the H2AX gene and the γH2AX signal in the epidermis by PCR (Supplementary Fig. 1A) and immunofluorescence on tissue sections (Supplementary Fig. 1B), respectively. Although the skin of the H2AX KO mice appeared macroscopically normal, when analysed microscopically, it displayed abnormalities. First, the KO epidermis was generally thicker than controls, with an increase in the number of cellular layers (Fig. 1A). Sebaceous glands of the KO skin appeared consistently larger. Basal cells and nuclei in the basal layer of the KO epidermis were larger than controls (Fig. 1B, arrows). Keratinocytes increase in cytoplasmic and nuclear size during differentiation [19, 20].

**Fig. 2:**
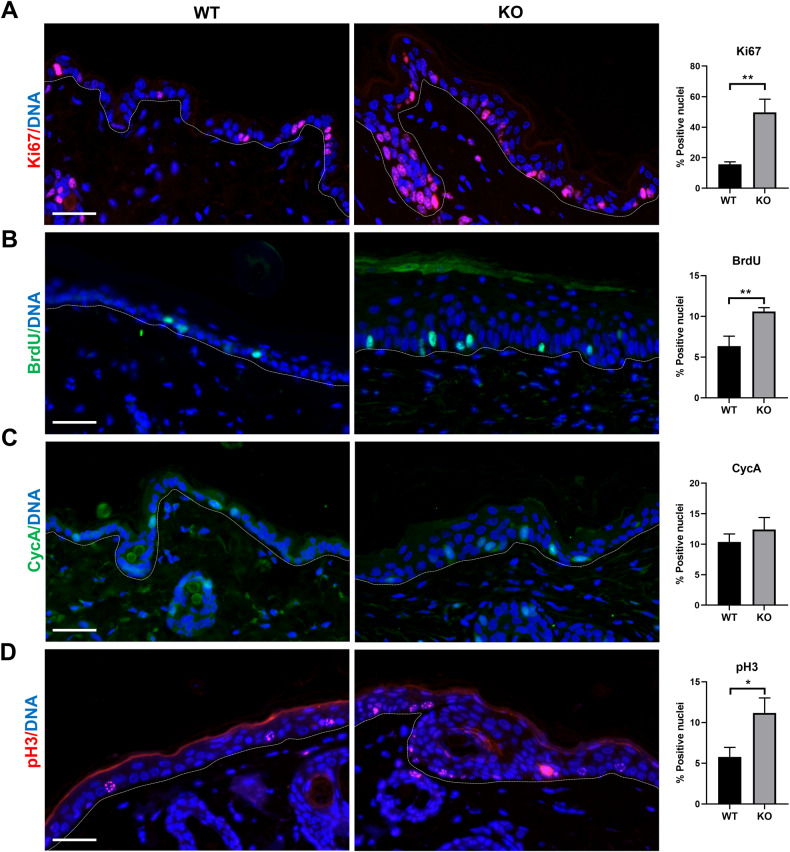
H2AX KO epidermis is more actively in cycle. **A–D** Representative immunofluorescence of H2AX WT or KO epidermis for Ki67 (**A**, red), BrdU (**B**, green), Cyclin A (**C**, green), pH3 (**D**, red). Note cells with a strong accumulation of pH3 in KO. Bar histograms represent the percent of basal positive nuclei for the molecules shown on the left. DNA in blue by DAPI. The broken line for the basement membrane. **p value <0.01, *p value <0.05. Student´s *t*-test. Data were mean ± SEM of five representative fields (more than 300 nuclei) of representative immunofluorescences. Scale bars: 20 µm.

To analyse the effect of the loss of H2AX on the epidermis, we labelled the skin for differentiating and cell cycle markers. First, we investigated the ratio of Ki67, a classical marker of cell cycle activity. KO epidermis contained a higher proportion of Ki67 positive cells than the WT controls (Fig. 2A). Consistently, KO epidermis displayed a higher index of BrdU incorporation (DNA replication; Fig. 2B). In addition, we stained the epidermis for Cyclin A, a key regulator of the S to G2 transition and for histone pH3, a marker of chromosomal condensation at metaphase (mitosis). Although less significant, the KO epidermis contained a higher proportion of cells positive for mitotic marker pH3, and although less significant, also for Cyclin A (Fig. 2C, D). Western blotting also showed an increase of Cyclin E1 and PCNA cell cycle proteins in the KO epidermis compared to controls (Supplementary Fig. 2A). Altogether, the results indicate that basal cells in the KO epidermis were more actively cycling. The distribution of cycling cells was also different in the KO epidermis. Positive cells were spread out and in groups, in contrast to the WT epidermis, where positive cells were more disperse. Altogether, the results show that basal cells within KO epidermis were more frequently in cycle.

**Fig. 3:**
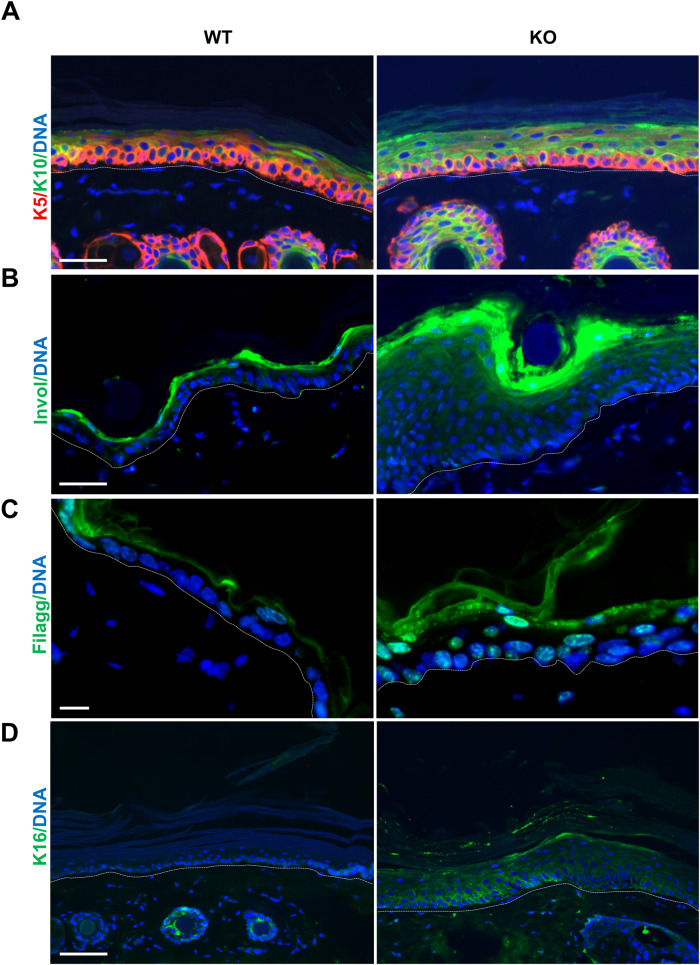
Loss of H2AX leads to increased differentiation (hyperkeratosis). **A–D** Representative immunofluorescence of H2AX WT or KO epidermis for keratin K5 (red) and K10 (green; **A**), involucrin (**B**, green), filaggrin (**C**, green), keratin K16 (**D**,green). DNA in blue by DAPI. Broken line for the basement membrane. Scale bars: 20 µm.

Keratinocytes block in G2/M upon deregulation of the cell cycle and replication stress, in response to irreparable damage. In turn, G2/M checkpoints trigger epidermoid terminal differentiation [15, 21]. While no significant differences in the expression of the basal marker Keratin K5, the expression of terminal differentiation markers Keratin K10 and involucrin started deeper and was more intense in KO than in the control epidermis (Fig. 3A, B). Filaggrin is a later marker that is normally expressed in the granular layer underneath the surface of the skin. The zone of Filaggrin expression within the epidermis was thickened and stronger in the KO epidermis and was also prematurely expressed in some nuclei of the basal layer (Fig. 3C). Analyses of markers of inflammation or senescence, interleukins IL-6 and IL-1β, TNF-α, or TGF-β showed no significant differences in the KO versus control epidermis (Supplementary Fig. 2B). The results altogether show that the H2AX KO epidermis cycles and differentiates faster and more markedly. Keratin K16, normally undetectable in normal epidermis [22] was extensively expressed in the KO tissue (Fig. 3D). K16 is a suprabasal keratin associated with skin hyperplasia and it is induced in post-mitotic epidermoid terminal differentiation [23].

**Fig. 4:**
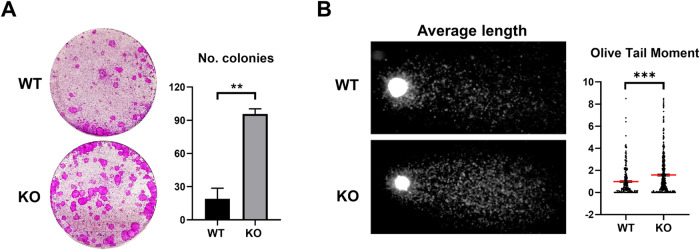
Loss of H2AX causes accumulation of DNA damage and an increase of proliferative progenitors. **A** Clonogenic capacity of cells from H2AX WT or KO epidermis as indicated. 10,000 total cells were plated per well in triplicates and stained 12 days after plated. Bar histogram displays the number of colonies obtained per well. **B** Nuclear DNA fragmentation as analysed by *comet* assay of freshly isolated keratinocytes in the WT or KO epidermis, as indicated. A scatter plot shows the quantitation of DNA damage according to the olive tail moment parameter. ***p value <0.001, **p value <0.01, Student´s *t*-test. Data were mean ± SEM of two animals per group (*n* = 2) and two independent experiments.

The above results altogether indicate that the lack of H2AX induces cell cycle hyperactivation. To assess for the proliferative capacity of epidermal cells, we isolated keratinocytes from the skin of H2AX KO or control mice and placed them in culture in vitro at low density, in stratifying conditions. As shown in Fig. 4A, cells from the KO epidermis had a higher capacity to form colonies than control cells. However, colonies reached the same size as controls and cells within the colonies differentiated completely at the same pace as normal cells (not shown). This suggests that although there were more cycling cells in the isolated pool, they had a limited capacity to proliferate as normal cells. We subsequently analysed the level of DNA damage in keratinocytes isolated from KO skin. As shown in Fig. 4B, KO cells displayed a higher level of DNA breaks, as measured by *comet* assays. A higher index of DNA damage was likely a consequence of the cell cycle deregulation and the impaired DNA repair due to the lack of γH2AX.

**Fig. 5:**
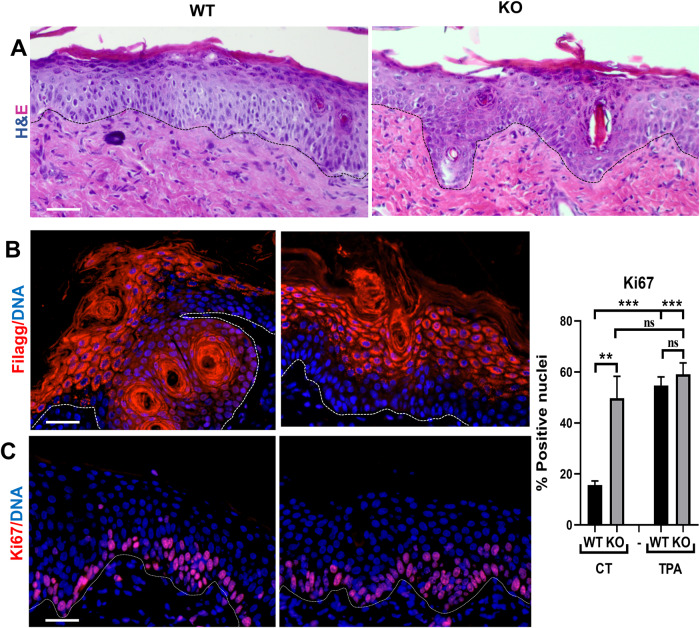
H2AX KO skin is constitutively activated. **A** Representative H&E staining of WT or KO back skin, treated for 7 days with either vehicle, or with TPA. **B** Representative immunofluorescence for late epidermoid differentiation marker Filaggrin (red) on sections of WT or KO skin untreated or treated with TPA for 7 days, as in **A**. DNA by DAPI (blue). **C** Representative immunofluorescence for cell cycle marker Ki67 (red) of the skin of animals, as in **A**. Broken line for the basement membrane. Bar histogram represents Ki67 positive cells of the epidermis as indicated. DNA in blue by DAPI. Scale bars: 20 µm. ***p value <0.001, **p value <0.01, ns not significant, one-way ANOVA, Tukey´s test. Data were mean ± SEM of five representative fields (more than 500 nuclei).

In order to see whether the defects in the skin in the absence of γH2AX were greater upon proliferative pressure, we challenged the epidermis by hyperactivating the cell cycle with 12-*O*-tetradecanoylphorbol 13-acetate (TPA). TPA is well known to induce epidermal hyperproliferation with hyperkeratosis (thickening of the differentiated strata) [24]. We treated the skin of control and KO mice with TPA for 7 or 10 days. In both cases, TPA induced a significant thickening of the epidermis (Fig. 5A and not shown). The difference in TPA was less clear in the KO epidermis. We labelled the skin of control and KO mice treated with TPA for differentiation and cell cycle markers. Whereas TPA induced a clear hyperplastic phenotype in the control epidermis with increased expression of Filaggrin (Fig. 5B). There was no significant differences between the control and the KO epidermis upon TPA. Therefore, the lack of H2AX did not enhance the effect of TPA. Consistently, the cell cycle marker Ki67, strikingly more frequent in untreated KO epidermis, displayed a similar proportion of positive cells in the mutant and the control tissue upon TPA (Fig. 5C).

The cellular DNA damage response, including γH2AX, is a cell cycle brake in response to DNA damage and protects cells from genomic instability. Therefore, it has been viewed as a potential tumour suppressor [7, 8]. In addition, we have observed loss of γH2AX in aggressive skin tumours [10]. Therefore, we aimed to study the effect of silencing H2AX in skin tumourigenesis. To this end, we subjected H2AX knock-out mice [4] to a well-established skin carcinogenesis protocol. This protocol administrates an initial topical treatment with the mutagen 7,12-dimethylbenz[a]anthracene (DMBA) and repeated doses of 12-*O*-tetradecanoylphorbol 13-acetate (TPA), as a tumour promotor, during several weeks, see materials and methods [25, 26]. The treatment typically generates skin papillomas or carcinomas.

**Fig. 6:**
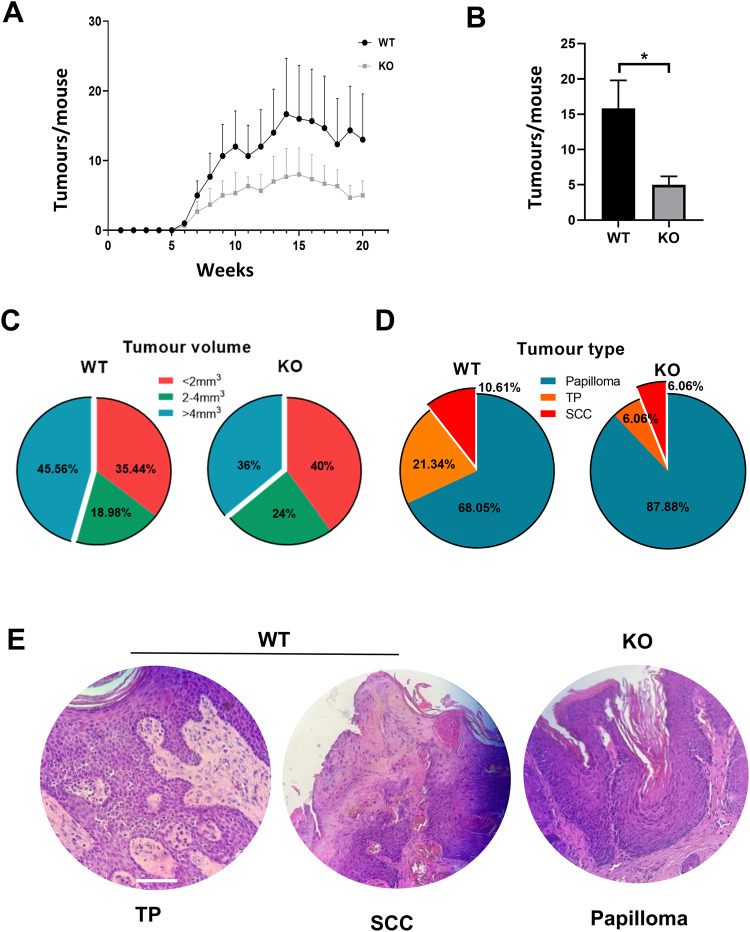
Loss of H2AX markedly impairs tumourigenesis. **A** Number of tumours per WT or KO mice observed during 20 weeks of the topical treatment with DMBA/TPA, as indicated. **B** Average number of tumours observed per WT or KO mice with time of treatment. **C** Percent of tumours according to their size (represented in units of volume). **D** Percent of type tumours developed according to their histological characteristics. **E** Representative H&E staining of transitional papilloma (TP) and squamous cell carcinoma developed in WT or a KO papilloma, as indicated. Scale bar: 50 µm. *p value <0.05, Student´s *t*-test. Data were mean ± SEM of four animals per group (*n* = 4) and two independent experiments.

As summarised in Fig. 6A, B and Supplementary Fig. 3, treatment of control littermates with DMBA/TPA caused an expected proportion of carcinomas in the skin, detected from week 6 onwards. However, the development of tumours in KO mice was significantly delayed (2 weeks). In addition, the average number of tumours per mice at the terminus was significantly smaller in the KO mice (5, versus 15 in control mice). In addition, the average size (Fig. 6C) and weight (4 mg versus 10 mg) of KO tumours at the terminus were strikingly reduced as compared to controls. Interestingly, these differences were found in males but not in females (not shown).

We next characterised the histology of tumours generated in the skin of control and KO littermates. As summarised in Fig. 6D, E, only 12,12% of the KO tumours were classified as SCC or transitional papillomas to SCC (TP), versus 31.95% in control mice. 87.88% of tumours were classified as papillomas in KO mice, versus 68.05% in control mice. Therefore, tumourigenesis was significantly compromised in the absence of H2AX.

**Fig. 7:**
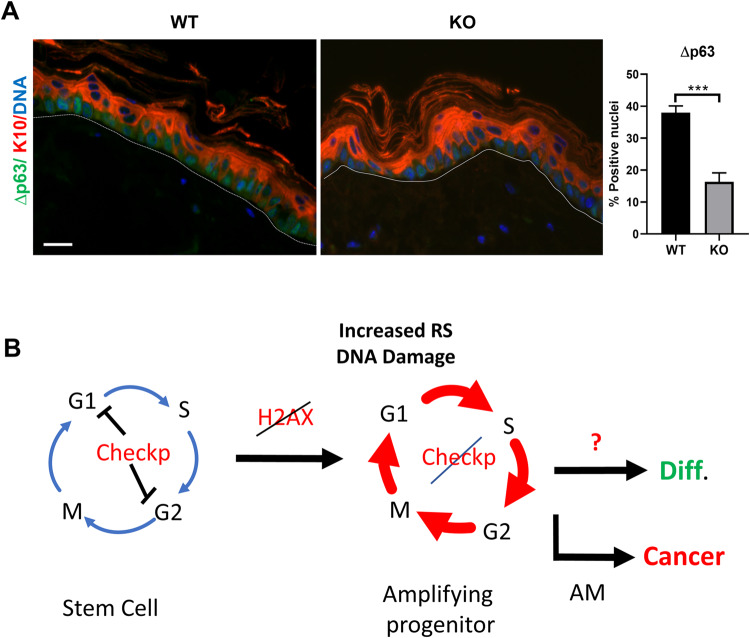
Loss of H2AX causes a reduction in the epidermal stem cell pool. **A** Representative double immunofluorescence for the epidermal stem cell marker ∆p63 (green) and differentiation marker Keratin K10 (red) in WT or KO mice, as indicated. DNA in blue by DAPI. Bar histogram represents the percent of ∆p63 positive basal nuclei cells of the epidermis. Broken line for the basement membrane. ***p value <0.001, Student´s *t*-test. Data were mean ± SEM of five representative fields (more than 500 nuclei). Scale bar: 20 µm. **B** Model for the induction of differentiation upon H2AX inactivation in the epidermis. In normal tissue, the lack of H2AX deregulates the cell cycle of stem cells, which leads to the accumulation of DNA damage due to increased replication stress (RS) and decreased DNA repair. Unknown signals recognise the damage and trigger terminal squamous differentiation (Diff.). This impairs the growth of rising tumour cells. Additional mutations in the signals blocking mitosis (AM) may lead to genomic instability and cancer progression.

The defects in epidermal homoeostasis and tumourigenesis in the H2AX KO mice might be due to impaired DNA repair and accumulation of mutations. This, together with the deregulation of the cell cycle in the absence of H2AX, might damage the stem cell population. To investigate this issue, we labelled the epidermis for a known marker of mouse epidermal stem cells, deltap63 (Δp63) [27]. As shown in Fig. 7A, Δp63 positive cells were strikingly more sparse and isolated in the KO epidermis compared to the control tissue.

## Discussion

γH2AX, through checkpoints, imposes a break to the cell cycle, in order to allow cells time for DNA repair [3, 4]. This is consistent with our observations that basal cells of the H2AX KO epidermis were more actively in cycle. This is in apparent contrast with Celeste et al (2002) [4]. They observed that H2AX KO MEFs underwent rapid senescence and cell cycle decay. However, cell cycle checkpoints in response to DNA damage trigger different cell fates depending on the cell type. Keratinocytes, upon replication stress and DNA damage are able to continue on the cell cycle, eventually blocking mitosis and triggering terminal differentiation [16]. In vivo, skin hyperplasia caused by cell cycle hyperactivation also typically results in hyperkeratosis (thickening of the differentiated layers) [16]. Replication stress-induced squamous differentiation might explain why hyperproliferation does not develop into tumourigenesis unless additional alterations break the differentiation signals. This was the phenotype that we found in the H2AX KO skin. Alleviating the γH2AX-induced checkpoints-brake accelerated cell cycle progression. Replication stress and impaired DNA repair was likely the cause for the higher index of DNA breaks found in epidermal cells. The lack of H2AX has been shown to reduce DNA repair without affecting cellular responses [28]. Celeste et al showed that H2AX is required for the congregation of proteins on the DNA lesion in order to repair it, but not for the detection of the DNA breaks. Therefore, alternative pathways must detect, and respond to, the higher level of DNA damage to induce epidermal differentiation in the absence of H2AX. TPA also causes skin hyperplasia with hyperkeratosis and this might be the reason why the phenotypes of control and KO mice were similar to TPA. We must conclude that TPA was unable to push the epidermal cell cycle further in the absence of H2AX.

As discussed above, H2AX contributes to the control of the cell cycle and to genomic stability and it has been proposed as a tumour suppressor and the gene locus was found deleted in a variety of cancers [7]. Therefore, our expectation was that the absence of the histone would enhance carcinogen-induced tumourigenesis. Instead, tumourigenesis was significantly reduced in time to develop, in the number of tumours and in their size and malignancy. Mouse skin chemical carcinogenesis is a well-characterised multi-step process that uses TPA to induce hyperplasia after initial mutations are caused by a single or a double treatment with carcinogen DMBA [24–26]. This model is well-established for recapitulating alterations in human cancer. It was previously shown that H2AX KO mice displayed minor evidence of spontaneous tumours unless tumour suppressor p53 was also silenced [4, 29]. p53 controls the cell cycle transitions, and in particular, it activates the DNA repair checkpoint [30]. The results above indicate that in the presence of p53 the loss of H2AX is not significantly carcinogenic. We therefore wanted to explore the consequences of subjecting cells without H2AX to a mutagenic pressure that might not be compensated by the p53 pathway. This, to our knowledge, has not been tested before.

Our results show not only that the absence of H2AX has no carcinogenic consequences, but also that epidermoid tumours need H2AX to develop. These tumours, as the normal self-renewal tissue, need the support of stem cells to grow. We obtained evidence within these lines. Stem cells need their cell cycle to be tightly controlled. As epidermal stem cells enter the phase of sustained proliferation, they quit their compartment and became progenitors. Epidermal progenitors, also referred to as *transit amplifying cells* (TAC) [31] or as *committed progenitors* (CP) [32], are committed to differentiate terminally after a few rounds of cell divisions. Highly proliferating keratinocytes lose cell cycle controls such as p53 or phospho-RB before they initiate the path to terminal post-mitotic differentiation [21, 33]. This causes DNA damage due to replication stress that, in turn, triggers antiproliferative terminal differentiation [16]. This is an anti-tumour mechanism that might prevent hyperproliferative skin during wound healing or psoriasis from tumourigenesis. The loss of H2AX caused hyperactivation of the cell cycle and impaired DNA repair in epidermal stem cells. This might limit their power to amplify and generate new tissue. This supports previous works suggesting a role for H2AX in stem cell biology [34–36].

The loss of epidermoid stem cells due to differentiation induced by cell cycle deregulation and DNA damage, might explain the reduction in carcinogenesis in the absence of H2AX. Tumour-initiating cells could not amplify further. KO tumours were clearly more differentiated than controls. We, therefore, conclude that loss of H2AX might contribute to cancer progression once tumour cells have lost the normal responses to DNA damage, but not before (Fig. 7B). Some malignant cells acquire the potential to bypass the cell cycle checkpoints in response to DNA damage and the DDR signals and to divide in spite of the damage with a high degree of genomic instability. Before these alterations appear, terminal differentiation prevails as a response to increased DNA damage in the absence of H2AX.

It must be noted that H2AX might also fulfil DNA damage-independent functions. For instance, such a role in chromosome segregation has been suggested [37, 38]. However, the basal hyperproliferation we have observed in the absence of H2AX does not argue for defective mitosis. In self-renewal tissues, it is critical that DNA is correctly repaired, especially in stem cells, and this would explain the existence of multiple or alternative molecules capable of detecting unrepaired DNA damage and then trigger differentiation. The challenge is to identify the molecules that recognise the DNA lesions and trigger the cellular responses in the absence of H2AX. These must be the drives of the strong anti-tumour mechanisms of self-renewal tissues whose alteration allows carcinogenesis.

## Materials and methods

### Transgenic mice

The study was approved by the Ethical Committee of Consejería de Ganadería, Pesca y Desarrollo Rural of the Gobierno de Cantabria (PI-05-20). Mice were sheltered in the Stabling and Animal Experimentation Service of the University of Cantabria (SEEA UC, Santander), a pathogen-free animal facility. Quality animal care and welfare standards were followed. Sick animals were euthanised in agreement with guidelines by endpoints in animal experimentation. All mice used in the studies were of the genetic background C57BL/6. Male mice 6-12 weeks after birth were used. H2AX knock-out (KO) mice were previously generated [4].

### In vivo treatments

For chemical skin carcinogenesis, the back skin of the mice, *H2AX* (+/+), *H2AX* (−/−) was shaved 48 h before the first 7,12-dimethylbenz[a]anthracene (DMBA, D-3254, Sigma-Aldrich) application. For initiation of carcinogenesis, two doses of DMBA were applicated. Then, these mice were treated topically in the dorsal skin with 200 µl DMBA (25 µg/200 µl acetone) by using a micropipette. For tumour promotion, 200 µl 12-*O*-Tetradecanoylphorbol 13-acetate (TPA, 4 µg/200 µl acetone; P1585, Sigma-Aldrich) were applicated in the back skin three times per week up to 20 weeks. Body weight, the number and size of tumours were recorded and documented weekly.

For hyperproliferation in the back or tail skin, *H2AX* (+/+), and *H2AX* (−/−), mice were treated topically for 7 or 10 days, respectively, with 100 µl TPA (100 µg/ml in 100% acetone) by using a micropipette.

For proliferation assay, intraperitoneal 5-Bromo-2′-deoxyuridine injection (BrdU, 50 mg/Kg weight in 100 µl 0.9% saline solution; H27260, Fisher) was applicated 1.5 h before euthanasia.

After the corresponding treatments, mice were sacrificed in a CO_2_ chamber. Tumours and back/tail skin were obtained.

### Isolating mouse keratinocytes

The isolation of mouse keratinocytes were modified from [39]. The tail was used from the mouse euthanised. The bone was removed from the tail tissue. The skin was rinsed once with 70% ethanol and washed with 1X PBS, Penicillin-Streptomycin (1:100) and Amphotericin (1:500) mix solution. Then, the skin was incubated in dispase II (25 units/ml; 04942078001, Roche) for 16 h at 4 °C. After this time, dispase was discarded and the epidermis was separated from the dermis with forceps. The epidermis once was washed with PBS. The epidermis was cut into small pieces and incubated in 0.25% trypsin-EDTA for 4 min at 37 °C in continuous shaking. Then, trypsin was blocked with Rheinwald FAD medium and epidermal cells were resuspended. Keratinocytes obtained were filtered through a 100 µM cell strainer. Cells were plated on tissue culture plates at a low density for clonogenicity assays (see below).

### Clonogenicity assays

For clonogenicity assays, 10,000 mouse keratinocytes were grown in high-calcium FAD medium and plated per triplicates in T6 wells. About 10–12 days later, the cultures were stained with rhodanile blue as described previously [20].

### Antibodies

The following antibodies were used: anti-Ki67 (ab16667, Abcam; IF), anti-BrdU (347580, Biosciences; IF), anti-filaggrin (PRB-417, Covance; IF), anti-involucrin (RINVOL, 924401, Biologend; IF), anti-K5 (SAB45016501, Sigma-Aldrich; IF), anti-K10 (sc-23877, Santa Cruz Biotechnology; IF), anti-K16 (sc-53255, Santa Cruz Biotechnology; IF), anti-Cyclin A (sc-751, Santa Cruz Biotechnology; IF), anti-pH3 (sc-8656-R, Santa Cruz Biotechnology; IF), anti-p-H2AX (sc-517348, Santa Cruz Biotechnology; IF), anti-p40 (deltaNp63, 24-8626-RBP1, ARP; IF), anti-Cyclin E1 (sc-248, Santa Cruz Biotechnology; WB), anti-PCNA (sc-56, Santa Cruz Biotechnology; WB) and anti-GAPDH (sc-32233, Santa Cruz Biotechnology; WB).The following secondary antibodies were used: Alexa Fluor® 488-conjugated goat anti-rabbit or anti-mouse IgG antibodies (Jackson; IF), Alexa Fluor® 594-conjugated goat anti-rabbit or anti-mouse IgG antibodies (Jackson; IF) and DyLight^TM^ 800-conjugated goat anti-mouse IgG antibody (Invitrogen; WB).

### Paraffin-embedded tissue, tissue histology and immunodetection

Tissues collected after euthanasia were placed in tissue cassettes and fixed in 10% neutral buffered formalin (HT501128, Sigma-Aldrich) for 24 h. Tissues were embedded in paraffin, cut it in sections of 3 µM and stained with Hematoxylin-Eosin staining according to the needs of the Biobanco of Hospital Universitario Marqués de Valdecilla (HUMV).

The thickness of the mouse epidermis was measured from the basal basement membrane to the surface of the skin of ten random fields of representative tissue sections using QuPath 0.3.0 (https://qupath.github.io/) software. The results were expressed in µm.

Immunofluorescence was modified from [40]. Paraffin was removed from the slides using xylene and the tissue was rehydrated with an ethanol gradient. Antigen retrieval tissue was performed in microwaving of slides in 0.01 M citrate buffer for 10 min. Sections of tissue were incubated in NH_4_Cl for 30 min at room temperature to reduce tissue autofluorescence. Then, sections of tissue were incubated with 8% goat serum for 1 h to block the Fc receptor. Primary and secondary antibodies were prepared in PBS-2% Bovine Serum Albumin (BSA) and incubated overnight at 4 °C and 1 h at room temperature, respectively.

For BrdU antigen retrieval, tissue was exposed to 2 M HCl for 20 min at room temperature for DNA denaturalisation. Then, HCl was neutralised with 0.1 M Sodium Tetraborate for 20 min at room temperature. The rest of the immunodetection protocol was the same as for other antigens (see above).

Quantitation of positive cells was performed by counting antigen-positive cells relative to total nuclei (DAPI staining) in the field. The nuclear size was quantified according to the area (in pixel^2^) using QuPath 0.3.0. Five representative fields were analysed from representative immunofluorescences. A minimum of 300 nuclei were analysed per mouse.

### Genotyping

Genotypic determination of mice was performed by extracting DNA from tail samples. Tail samples were incubated overnight at 55 °C with 400 µL lysis buffer (10 mM Tris pH 8.0, 100 mM NaCl, 0.5% SDS, 50 mM EDTA pH 8.0) and 20 µL of proteinase K (20 mg/mL; EO0492, Thermo Scientific). Samples were vortexed vigorously for 2 min and 300 µL of lysis buffer were added. About 250 µL of 6 M NaCl were added and the mix was vortexed for 2 min. The samples were centrifugated at 13,000 rpm for 10 min at RT. The supernatant was then separated in a clean tube. 500 µL of isopropanol were added, and the solution was vortexed for 2 min. Samples were centrifugated at 13,000 rpm for 10 min at 4 °C and the supernatant was discarded. 1 mL of 70% ethanol was added, and the samples were centrifugated at 13,000 rpm for 5 min at 4 °C. The supernatant was discarded, and the samples were left to dry for 1 h at RT. The DNA was resuspended in 500 µL of TE buffer (10 mM Tris pH 8.0, 0.1 mM EDTA pH 8.0) and incubated for 20 min at 55 °C to eliminate remaining RNA. The DNA was quantified in Nanodrop (NanoDrop One, Thermo Scientific) and was used as template in the polymerase chain reaction (PCR). Primers used were [4]: HX5 (WT and KO: 5´- CTCTTCTACCTCGTACACCATGTCCG −3´); HX3 (WT: 5´- CGAAGTGGCTCAGCTCTTTCTGTGAGGG −3´ and KXR (KO: 5´- GTCACGTCCTGCACGACGCGAGC −3´).

### Western blotting

Epidermal keratinocytes were isolated from tail mouse skin as above. Cell pellets were incubated in lysis buffer (150 mM NaCl, 50 mM Tris pH 8, 20 mM NaF, 1% NP40, 1 mM EDTA pH 8, 0.2% SDS 1 and supplemented with phosphatase (A32957, Thermo Scientific) and protease (539131, Calbiochem) inhibitors) for 10 min. Then, cells were mixed by pipetting and digested by two rounds of 5 min sonication. Cells were centrifuged at 12,000 rpm for 15 min at 4 °C. The supernatant was collected and total protein quantification was carried out by a fluorometric system Qubit 4.0 (Life Technologies). Samples were separated by SDS-PAGE and transferred to nitrocellulose membranes. After blocking and incubating with primary (overnight, 4 °C) and secondary (1 h, room temperature) antibodies, immunocomplexes were finally detected with an Odyssey Infrared-Imaging System (Li-Cor Biosciences). Original blots are shown in Supplemental Material.

### Reverse transcriptase-quantitative polymerase chain reaction (RT-PCR)

Epidermal keratinocytes were isolated from mouse tail skin as above. Total RNA was isolated from cell pellets and reverse-transcribed using the NucleoSpin® RNA kit (740955.50, Macherey-Nagel) and the iScript™ cDNA synthesis kit (4106228, Bio-Rad) according to the manufacturer’s instructions. The cDNA was amplified by real-time PCR using iQ™ SYBR Green supermix (1708880, Bio-Rad) and CFX Connect^TM^ Real-Time PCR Detection System (Bio-Rad). Results were normalised to β-actin levels. Primers used in this study for mouse genes were: IL-1β (5′- TCGCTCAGGGTCACAAGAAA −3′ and 5′- CATCAGAGGCAAGGAGGAAAAC −3′), IL-6 (5′- CAACCACGGCCTTCCCTACT −3′ and 5′- TTCTGCAAGTGCATCATCGTTGT −3′), TNF-α (5′- TCGTAGCAAACCACCAAGTG −3′ and 5′- AGATAGCAAATCGGCTGACG −3′), TGF-β (5′- TTGCCCTCTACAACCAACACAA −3′ and 5′- GGCTTGCGACCCACGTAGTA −3′) and β-Actin (5′- CCTTCTTGGGTATGGAATCC −3′ and 5′- ATCTTCATGGTGCTAGGAGC-3′).

### Statistical analyses

H/E staining or immunofluorescence was randomly selected for the analyses. Data were presented as mean ± SEM from at least two mice in freshly isolated keratinocytes (*n* = 2), and two independent experiments, as shown in each figure legend. Statistical methods are appropriate for every figure, relative to the normal mouse groups, for estimated variations and similarity among groups. Data sets were compared using an unpaired two-tailed Student’s *t*-test when two data sets or One-way ANOVA when more than two data sets were analysed (GraphPad Prism version 8.0.1). For multiple comparison, the Tukey test was used. A *P* value of less than 0.05 was considered statistically significant. Control and sample mice were chosen when possible to be littermates or at least of the same age. All animal samples analysed within the same group displayed similar results. Mice within each group were chosen by homogenous genotype, blind to the results.

### Supplementary information

Supplementary Figures

Full length blots

## Data Availability

All data and materials are presented in the main manuscript or additional supporting files.

## References

[1] Lehmann AR, McGibbon D, Stefanini M. Xeroderma pigmentosum. Orphanet J Rare Dis. 2011;6:70. 10.1186/1750-1172-6-70 PMC3221642 22044607

[2] Blackford AN, Jackson SP (2017). ATM, ATR, and DNA-PK: the trinity at the heart of the DNA damage response. Mol Cell Cell Press.

[3] Rogakou EP, Pilch DR, Orr AH, Ivanova VS, Bonner WM. DNA double-stranded breaks induce histone H2AX phosphorylation on serine 139. J Biol Chem. 1998;273:5858–68. 10.1074/jbc.273.10.5858 9488723

[4] Celeste A, Petersen S, Romanienko PJ, Fernandez-Capetillo O, Chen HT, Sedelnikova OA (2002). Genomic instability in mice lacking histone H2AX. Science.

[5] Fernandez-Capetillo O, Lee A, Nussenzweig M, Nussenzweig A (2004). H2AX: the histone guardian of the genome. DNA Repair.

[6] Hanahan D, Weinberg RA (2011). Hallmarks of cancer: the next generation. Cell.

[7] Bonner WM, Redon CE, Dickey JS, Nakamura AJ, Sedelnikova OA, Solier S (2008). γH2AX and cancer. Nat Rev.

[8] Srivastava N, Gochhait S, de Boer P, Bamezai RNK (2009). Role of H2AX in DNA damage response and human cancers. Mutat Res.

[9] Wang B, Zhang Z, Xia S, Jiang M, Wang Y (2019). Expression of γ-H2AX and patient prognosis in breast cancer cohort. J Cell Biochem.

[10] Alonso-Lecue P, de Pedro I, Coulon V, Molinuevo R, Lorz C, Segrelles C (2017). Inefficient differentiation response to cell cycle stress leads to genomic instability and malignant progression of squamous carcinoma cells. Cell Death Dis.

[11] Palla VV, Karaolanis G, Katafigiotis I, Anastasiou I, Patapis P, Dimitroulis D (2017). gamma-H2AX: can it be established as a classical cancer prognostic factor?. Tumor Biol.

[12] Rezaeian AH, Li CF, Wu CY, Zhang X, Delacerda J, You MJ (2017). A hypoxia-responsive TRAF6-ATM-H2AX signalling axis promotes HIF1α activation, tumorigenesis and metastasis. Nat Cell Biol.

[13] Celeste A, Difilippantonio S, Difilippantonio MJ, Fernandez-Capetillo O, Pilch DR, Sedelnikova OA (2003). H2AX haploinsufficiency modifies genomic stability and tumor susceptibility. Cell.

[14] Bassing CH, Suh H, Ferguson DO, Chua KF, Manis J, Eckersdorff M (2003). Histone H2AX: a dosage-dependent suppressor of oncogenic translocations and tumors. Cell.

[15] Sanz-Gómez N, de Pedro I, Ortigosa B, Santamaría D, Malumbres M, de Cárcer G (2020). Squamous differentiation requires G2/mitosis slippage to avoid apoptosis. Cell Death Differ.

[16] Molinuevo R, Freije A, Contreras L, Sanz JR, Gandarillas A (2020). The DNA damage response links human squamous proliferation with differentiation. J Cell Biol.

[17] Gandarillas A (2012). The mysterious human epidermal cell cycle, or an oncogene-induced differentiation checkpoint. Cell Cycle..

[18] San Juan L, Freije A, Sanz-Gómez N, Jiménez-Matías B, Pleguezuelos-Manzano C, Sanz JR (2023). DNA damage triggers squamous metaplasia in human lung and mammary cells via mitotic checkpoints. Cell Death Discov.

[19] Banks-Schlegel S, Green H (1981). Involucrin synthesis and tissue assembly by keratinocytes in natural and cultured human epithelia. J Cell Biol.

[20] Zanet J, Freije A, Ruiz M, Coulon V, Sanz JR, Chiesa J (2010). A mitosis block links active cell cycle with human epidermal differentiation and results in endoreplication. PLoS ONE.

[21] Freije A, Ceballos L, Coisy M, Barnes L, Rosa M, De Diego E (2012). Cyclin e drives human keratinocyte growth into differentiation. Oncogene.

[22] Leigh IM, Navsaria H, Purkis PE, McKay IA, Bowden PE, Riddle PN (1995). Keratins (K16 and K17) as markers of keratinocyte hyperproliferation in psoriasis in vivo and in vitro. Br J Dermatol.

[23] Sanz-Gómez N, Freije A, Gandarillas A (2020). Keratinocyte differentiation by flow cytometry. Methods Mol Biol.

[24] Furstenberger G, Berry DL, Sorg B, Marks F. Skin tumor promotion by phorbol esters is a two-stage process. Proc Natl Acad Sci USA. 1981;78:7722–26. 10.1073/pnas.78.12.7722 PMC349342 6801661

[25] Abel EL, Angel JM, Kiguchi K, DiGiovanni J (2009). Multi-stage chemical carcinogenesis in mouse skin: fundamentals and applications. Nat Protoc.

[26] Huang PY, Balmain A (2014). Modeling cutaneous squamous carcinoma development in the mouse. Cold Spring Harb Perspect Med.

[27] McKeon F (2004). p63 and the epithelial stem cell: more than status quo?. Genes Dev.

[28] Celeste A, Fernandez-Capetillo O, Kruhlak MJ, Pilch DR, Staudt DW, Lee A, et al. Histone H2AX phosphorylation is dispensable for the initial recognition of DNA breaks. Nat Cell Biol. 2003;5:675–9. 10.1038/ncb1004 12792649

[29] Bassing CH, Suh H, Ferguson DO, Chua KF, Manis J, Eckersdorff M, et al. Histone H2AX: a dosage-dependent suppressor of oncogenic translocations and tumors. Cell. 2003;114:359–70 10.1016/s0092-8674(03)00566-x 12914700

[30] Aylon Y, Oren M (2011). P53: guardian of ploidy. Mol Oncol.

[31] Zhang B, Hsu YC. Emerging roles of transit-amplifying cells in tissue regeneration and cancer. Wiley Interdisc. Rev. Dev. Biol. 2017;6:10.1002/wdev.282. 10.1002/wdev.282 PMC5561490 28670819

[32] Jones P, Simons BD (2008). Epidermal homeostasis: do committed progenitors work while stem cells sleep?. Nat Rev.

[33] Dazard JE, Piette J, Basset-Seguin N, Blanchard JM, Gandarillas A (2000). Switch from p53 to MDM2 as di€erentiating human keratinocytes lose their proliferative potential and increase in cellular size. Oncogene.

[34] Andäng M, Hjerling-Leffler J, Moliner A, Lundgren TK, Castelo-Branco G, Nanou E (2008). Histone H2AX-dependent GABAA receptor regulation of stem cell proliferation. Nature.

[35] Turinetto V, Orlando L, Sanchez-Ripoll Y, Kumpfmueller B, Storm MP, Porcedda P (2012). High basal γH2AX levels sustain self-renewal of mouse embryonic and induced pluripotent stem cells. Stem Cells.

[36] Zhao B, Tan TL, Mei Y, Yang J, Yu Y, Verma A (2016). H2AX deficiency is associated with erythroid dysplasia and compromised haematopoietic stem cell function. Sci Rep.

[37] Ichijima Y, Sakasai R, Okita N, Asahina K, Mizutani S, Teraoka H (2005). Phosphorylation of histone H2AX at M phase in human cells without DNA damage response. Biochem Biophys Res Commun.

[38] McManus KJ, Hendzel MJ (2005). ATM-dependent DNA damage-independent mitotic phosphorylation of H2AX in normally growing mammalian cells. Mol Biol Cell.

[39] Guinea-Viniegra J, Zenz R, Scheuch H, Jiménez M, Bakiri L, Petzelbauer P (2012). Differentiation-induced skin cancer suppression by FOS, p53, and TACE/ADAM17. J Clin Investig.

[40] Segrelles C, Moral M, Fernanda Lara M, Ruiz S, Santos M, Leis H (2006). Molecular determinants of Akt-induced keratinocyte transformation. Oncogene.

