# Supplementary material for: DNA damage signalling histone H2AX is required for tumour growth
Source: Cell Death Discov. 2024 Feb 24;10:99. doi: 10.1038/s41420-024-01869-9 (PMC10894207; doi:10.1038/s41420-024-01869-9)
Supplement: Supplementary file 1 — Supplementary Figures [file 41420_2024_1869_MOESM1_ESM.pdf]

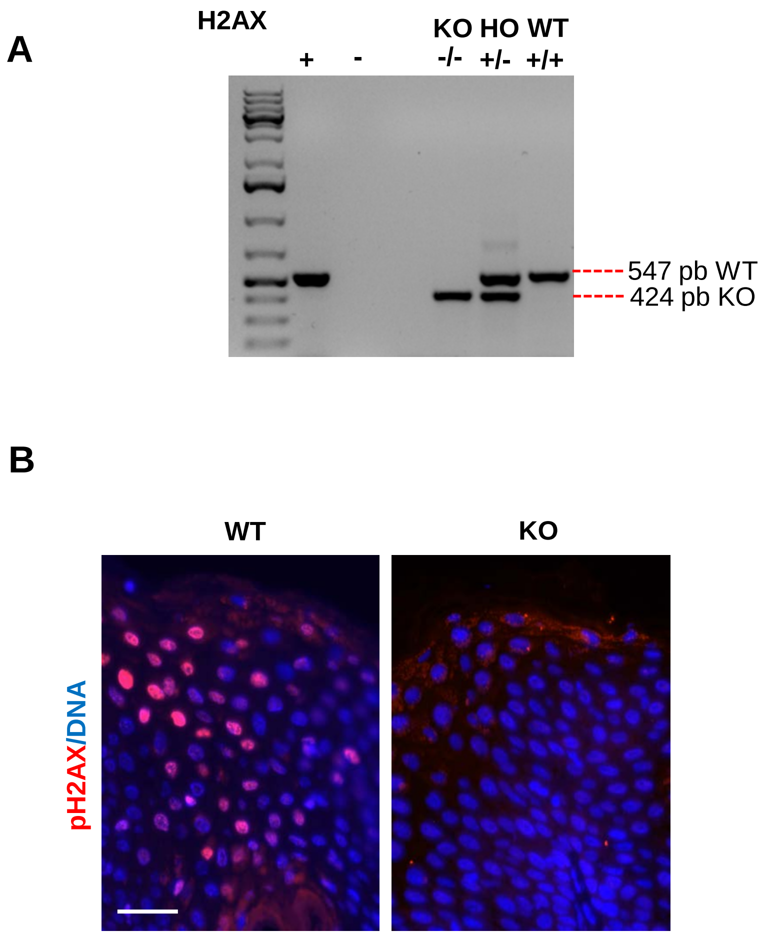


**Contreras et al**

**Supplementary Figure 1. Deletion of the H2AX gene in KO mice. A.** Electrophoresis of PCR for the deletion of *H2AX* gene in tail sample. +, positive founder, wild type (WT) control; -, negative, knock-out (KO) founder control. Example of the progeny: +/+, WT mice; +/-, heterozygous mice (HO); -/-, KO. **B.** Representative immunofluorescence for γH2AX (red) in WT or KO tumours. DNA by DAPI (blue). Scale: 20 µm.

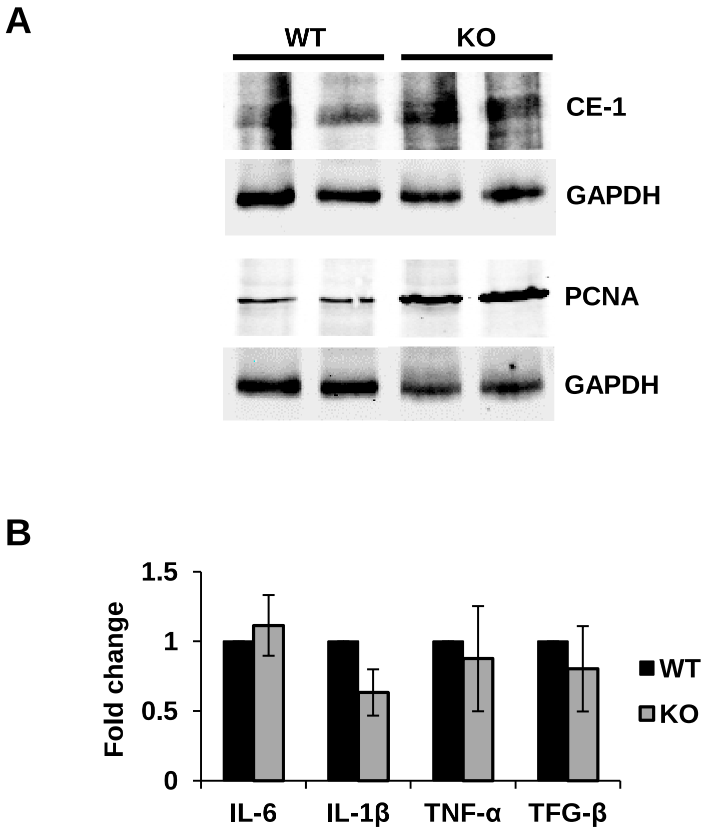


**Contreras et al**

**Supplementary Figure 2. Expression of cell cycle, inflammation or senescence markers in WT or KO epidermis. A.** Cyclin-E1 (CE-1) or PCNA protein expression by Western blotting in H2AX WT or KO mouse tail epidermis. Glyceraldehyde 3-phosphate dehydrogenase (GAPDH) is used as loading control. **B.** mRNA fold change of inflammation and senescence markers interleukins IL-6 and IL-1β, TNF-α or TNAF-β, by RT-PCR in H2AX wild type (WT) and knock-out (KO) mouse tail epidermis. Data are mean ± SEM of five animals per group.

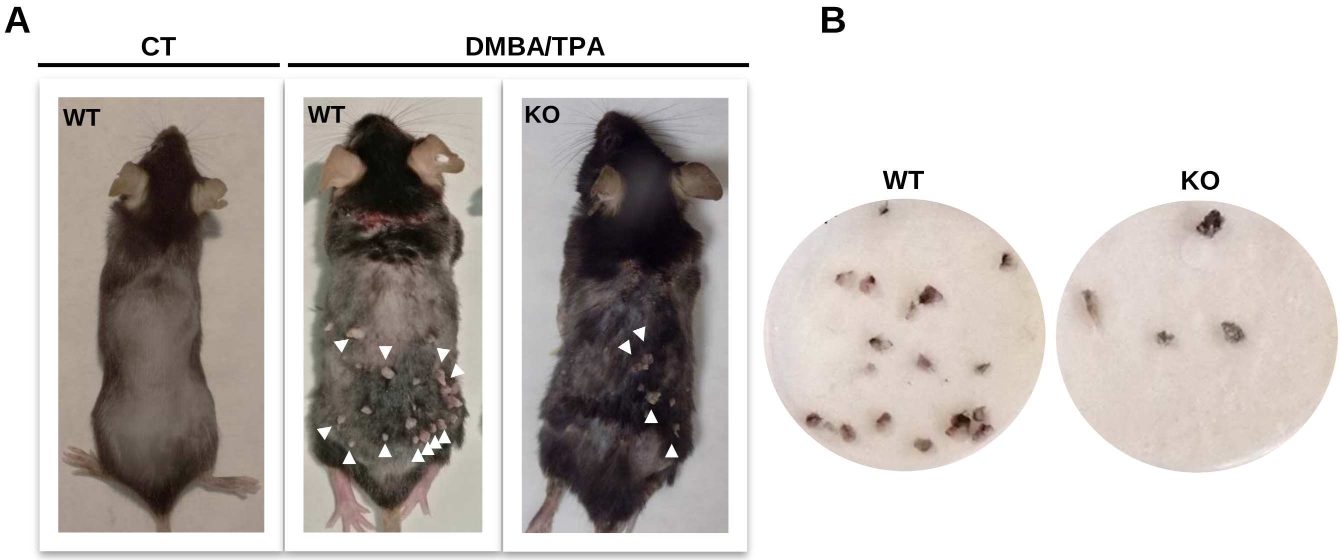


Contreras et al

**Supplementary Figure 3. Tumours induced on the skin of WT or KO mice. A.** Representative photographs of WT or KO mice with skin tumours developed 20 weeks after topical treatment with DMBA/TPA. **B.** Photographs of all the tumours per mouse after removal. CT: WT mouse treated with the acetone vehicle only.
