# Supplementary figures and images for: DNA damage signalling histone H2AX is required for tumour growth
Source: Cell Death Discov. 2024 Feb 24;10:99. doi: 10.1038/s41420-024-01869-9 (PMC10894207; doi:10.1038/s41420-024-01869-9)

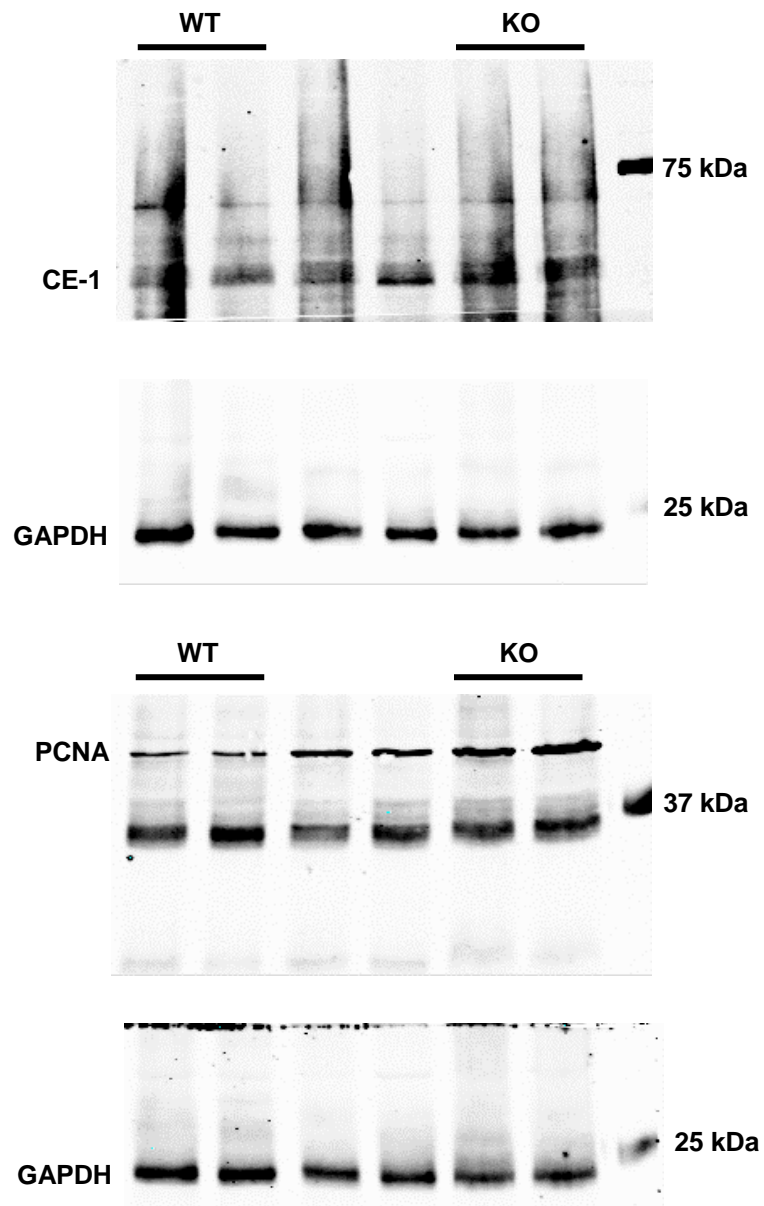

Contreras et al

Original western blots in Supplementary Figure 2.

Supplement: Supplementary file 2 — Full length blots [file 41420_2024_1869_MOESM2_ESM.pdf]
